# An investigation into aflatoxin M_**1**_ in slaughtered fattening pigs and awareness of aflatoxins in Vietnam

**DOI:** 10.1186/s12917-017-1297-8

**Published:** 2017-11-28

**Authors:** Hu Suk Lee, Johanna Lindahl, Hung Nguyen-Viet, Nguyen Viet Khong, Vuong Bui Nghia, Huyen Nguyen Xuan, Delia Grace

**Affiliations:** 1 International Livestock Research Institute, Regional Office for East and Southeast Asia, Room 301-302, B1 Building, Van Phuc Diplomatic Compound, 298 Kim Ma Street, Ba Dinh District, Hanoi, Vietnam; 2 International Livestock Research Institute, Nairobi, Kenya; 3 Swedish University of Agricultural Sciences, Uppsala, Sweden; 4 National Institute of Veterinary Research, 86 Truong Chinh, Phuong Mai, Dong Da, Hanoi, Vietnam

**Keywords:** Vietnam, ELISA, Pig, Urine, Aflatoxins, Survey, Perception/knowledge

## Abstract

**Background:**

Aflatoxin M_1_ (AFM_1_) is a hydroxylated metabolite formed after aflatoxin B_1_ (AFB_1_) is consumed by humans and animals; it can be detected in urine, milk and blood. It is well recognized that AFB_1_ is toxic to humans and other animals. The International Agency for Research on Cancer (IARC) classifies aflatoxins as group 1 carcinogens and AFM_1_ as group 2B carcinogen. The main objective of this study was to evaluate the exposure of pigs to aflatoxins as well as to assess the public awareness of aflatoxins among people in five provinces in Vietnam.

**Results:**

A total of 1920 urine samples were collected from slaughterhouses located in five provinces. Overall, the positive rate of AFM_1_ was 53.90% (95% confidence interval 51.64–56.15) using a cut-off of 0.15 μg/kg (range: limit of detection to 13.66 μg/kg, median: 0.2 μg/kg and mean: 0.63 μg/kg). A total of 252 people from the general population were interviewed from 5 provinces, and overall 67.86% reported being aware of aflatoxins. We also found that men and more highly educated had significantly increased awareness of aflatoxins compared to the females and primary/secondary school group. The respective odds ratios (ORs) were as follows: “male” group (OR: 2.64), “high school educated” group (OR: 3.40) and “college/university or more educated” group (OR: 10.20).

**Conclusions:**

We can conclude that pigs in Vietnam are exposed to aflatoxins to varying degrees, and there may be a risk that pork products could contain AFM_1_. Further investigation is needed into the possible health impacts as well as to aid in establishing regulations for animal feed to reduce the health impacts in humans and animals.

## Background

Aflatoxins are natural toxic metabolites of *Aspergillus* spp. (*A. flavus* and *A. parasiticus*) [1–3]; they may occur in a wide range of food commodities, and some, such as peanuts, maize and nuts are especially prone to contamination [4–6]. Aflatoxin M_1_ (AFM_1_) is a hydroxylated metabolite of aflatoxin B_1_ (AFB_1_) produced in humans and other animals that consume contaminated food, and can be detected in urine, milk and blood [7–10]. It is well recognized that AFB_1_ is toxic to humans and other animals. The International Agency for Research on Cancer (IARC) classifies aflatoxins as group 1 carcinogens and AFM_1_ as group 2B carcinogen [11–14]. Human exposure to aflatoxins can occur via consumption of agricultural products (such as maize, rice, peanuts and nuts etc.) or following consumption of dairy products (such as milk, cheese and yoghurt), meat and eggs produced by livestock exposed to aflatoxins [15–17]. Long-term exposure to aflatoxins is a major risk factor for liver cancer [18]. In animals, chronic exposure to aflatoxins is associated with weight loss and reproductive problems [19–21]. Particularly in pigs, the associated clinical signs are lethargy, hypothermia and icterus [22–24]. The main effect of aflatoxin exposure in pigs, however, is reduction in feed intake and average daily weight gain [19, 25, 26].

Vietnam is a tropical country which is hot and humid, providing favorable conditions for fungal growth [27, 28]. Some studies have been conducted to assess AFB_1_contamination in agricultural products in Vietnam. One study found AFB_1_ in 83.3–100% of pig feed products [29]. Other studies reported AFB_1_ in rice, cassava and maize [4, 28, 30].

However, to our knowledge, few studies have been conducted to evaluate the concentrations of AFM_1_ in pig urine in Vietnam as well as to assess the perception and knowledge of aflatoxins. One study in two pigs found only up to 16% of a dose of AFB_1_ fed to the animals could be detected in the urine [31]. In another pig trial in Vietnam, approximately 23% of ingested AFB_1_ was converted to AFM_1_ and excreted in the urine [32]. Another older study found that the average AFM_1_ concentration in pig urine was 2.29 ng/ml after feeding 12.7 μg/kg AFB_1_ over 12 weeks [33]. Therefore, the main objective of this study was to evaluate the concentrations of AFM_1_ in the urine of pigs slaughtered for human consumption and to assess public awareness of aflatoxins among the general population in five provinces in Vietnam.

## Methods

### Study locations and data collection

**Fig. 1:**
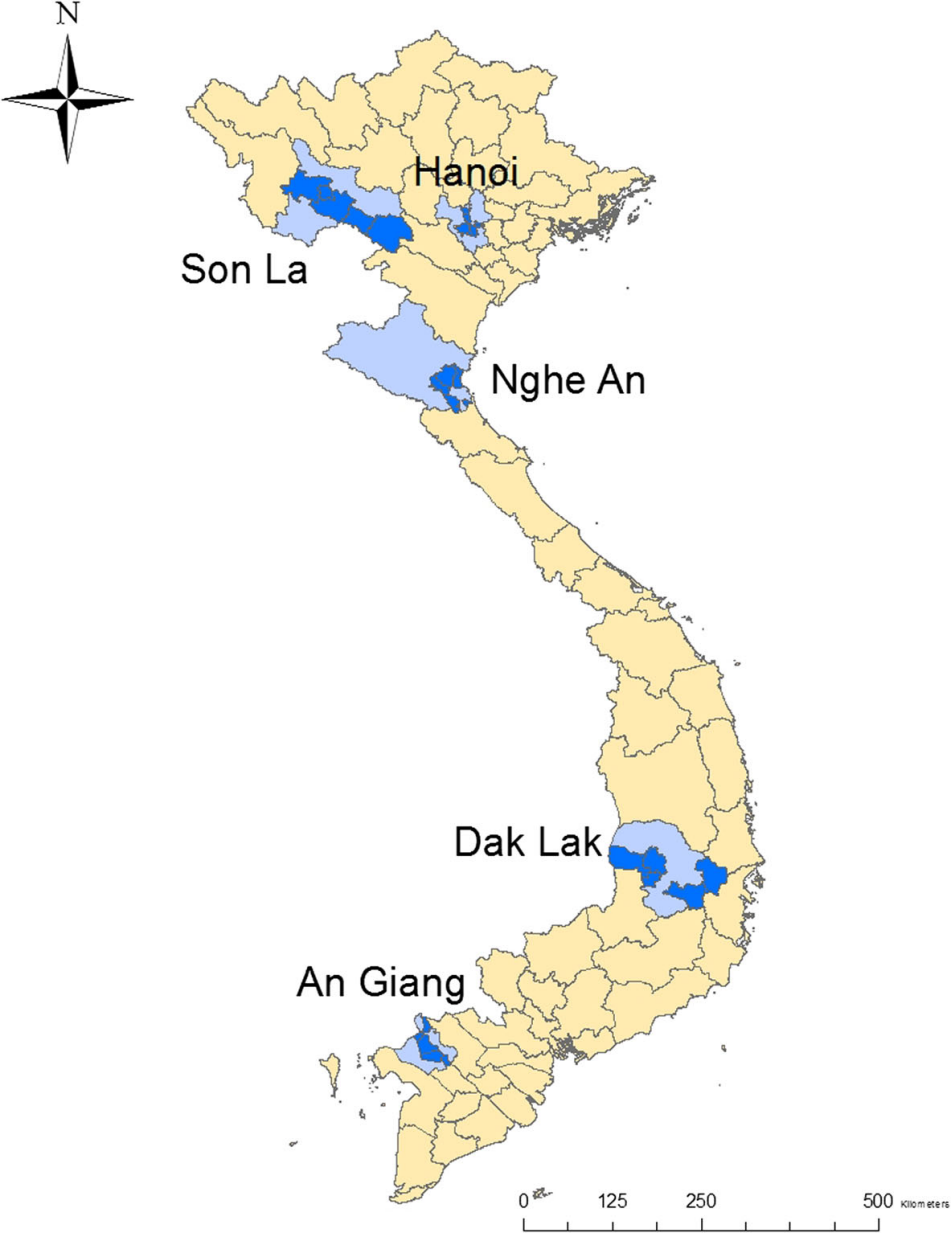
Selected sampling districts (dark blue) within each province (light blue)

Vietnam’s climate shows much variation because of its geography (Fig. 1). According to the Köppen-Geiger classification, the climate of southern Vietnam is mainly ‘tropical wet dry’, northern Vietnam has a “humid subtropical” climate and most of the middle and the extreme south are “tropical monsoonal” (Table 1) [34]. Vietnam is commonly divided into eight ecological zones based on geographical features and climatic conditions [35]. Here, the provinces were selected based on high maize production and to represent different ecological and climatic zones.

**Table 1:** Climate classification and ecological region of each province

Province	Köppen climate classification	Ecological region
Hanoi	Cw: Humid subtropical	Red River Delta
Son La	Cw: Humid subtropical	North West
Nghe An	Aw: Tropical wet dry, and Am: tropical monsoonal	North Central Coast
Dak Lak	Aw: tropical wet dry	Central Highlands
An Giang	Aw: Tropical wet dry	Mekong River Delta

Urine samples were collected from slaughtered fattening pigs (mostly 6–9 months old and weighing 60–120 kg; 11 pigs were out of range) in five provinces (Son La, Hanoi, Nghe An, Dak Lak and An Giang) between January and early June 2016. The sample size was calculated based on 50% prevalence, a precision level of 5% and 95% confidence interval. At least 385 samples per province were collected using multi-stage sampling (province-district-commune). For each province, a total of 25 communes (5 communes per district) were selected from 5 districts based on the availability of pig slaughterhouses. Within the commune, 15–16 samples were randomly collected from more than one slaughterhouse (Fig. 1). Before collecting the samples, it was confirmed that the pigs had been raised in the selected province only. Data were collected on the sex and breed of the pigs. In communes where pigs were sampled, households and pedestrians close to the slaughterhouses were selected by convenience sampling and interviewed in Vietnamese to assess their awareness of aflatoxins.

### Laboratory analysis

All urine samples were immediately placed in cool boxes at the slaughterhouses and stored at −20 °C at local laboratories until transportation to the National Institute of Veterinary Research (NIVR) in Hanoi where they were analyzed. Prior to the analysis, all samples were thawed and centrifuged at 3000 g for 5 min to eliminate debris and then supernatant was used for the determination of AFM_1_ levels. The concentration of AFM_1_ was determined using a commercially available enzyme-linked immunosorbent assay (ELISA) (Helica Biosystems Inc., Santa Ana, CA. USA). This commercial ELISA has been specifically developed and validated for urine testing, and has been used in many previous studies. We followed all the procedures based on the protocol provided by the manufacturer. Finally, the optical density (OD) of the sample was compared to a standard curve, and then each sample level (μg/kg) was determined based on the regression equation. The standard curve covers 150–4000 ppt. A cut-off level of 0.15 μg/kg [(limit of detection (LOD) determined by the manufacturer] was used for calculating the proportion of positive samples. In addition, the mean, median and range were investigated for each province based on samples with AFM_1_ concentrations above 0.15 μg/kg.

### Data analysis

A logistic regression model was developed to assess the association between the demographic variables (sex and breed) and positive status (≥LOD) while province was included as a random effect in the model.

For the awareness study, demographic information was collected via questionnaires [variables: age group (<20 years, 20–29 years, 30–39 years, 40–49 years, 50–59 years and ≥60 years), gender (male and female), education level (none, primary/secondary school, high school and college/university or more) and occupation (farmers, retailers, feed manufactures and others)]. A multivariable logistic regression model was used to evaluate the association between the demographic variables and awareness of aflatoxins as the dependent variable (question: *Have you heard about aflatoxins?*). For variable screening, chi-square tests were conducted for each variable, with only significant variables included in the final model. In addition, province was treated as a random effect to account for clustering. The final model fit was assessed using the Hosmer-Lemeshow test [36]. Variables with *p*-values <0.05 were set for statistical significance in the model. Odds ratio (OR) and 95% confidence interval (CI) were calculated by exponentiation of the coefficients from the regression model. All data were entered into Microsoft Excel 2013 and analyzed using STATA (version 14.0, StataCorp, College Station, TX, USA). ArcGIS version 10.3.1 ArcMap (ESRI, Redlands, CA, USA) was used to generate the map.

## Results

A total of 1920 urine samples were collected from slaughterhouses located in five provinces (Hanoi: *n* = 385, Son La: *n* = 383, Nghe An: *n* = 375, Dak Lak: *n* = 384 and An Giang: *n* = 393). Overall, the positive rate of AFM_1_ was 53.90% (95% CI 51.64–56.15) using a cut-off of 0.15 μg/kg (range: LOD to 13.66 μg/kg, median: 0.2 μg/kg and mean: 0.63 μg/kg) (Table 2). Son La and Hanoi had significantly higher positive rates, whereas An Giang had a significantly lower positive rate compared to other provinces.

**Table 2:** Distribution of aflatoxin M_1_ levels in pigs from five provinces in Vietnam

Province (No.)	Samples above LOD (% with 95% CI)^a^	Mean (μg/kg)^a^	Median (μg/kg)^a^	Range (μg/kg)^a^
Hanoi (385)	292 (75.84, 95% CI 71.25–80.04)	0.41	0.19	<LOD - 8.05
Son La (383)	316 (82.51, 95% CI 78.32–86.18)	1.23	0.32	<LOD - 7.35
Nghe An (375)	245 (65.33, 95% CI 60.28–70.15)	0.24	0.18	<LOD - 1.42
Dak Lak (384)	167 (43.49, 95% CI 38.47–48.61)	0.50	0.18	<LOD - 13.66
An Giang (393)	15 (3.82, 95% CI 2.15–6.22)	0.19	0.17	<LOD - 0.30
Total (1920)	1035 (53.90, 95% CI 51.64–56.15)	0.63	0.20	<LOD - 13.66

^a^Mean and median were calculated from the samples above limit of detection (LOD ≥ 0.15 μg/kg)

From collected demographic information (breed and sex), we found that female pigs were significantly more likely to have AFM_1_ (OR: 1.45, 95% CI: 1.08–1.96) in their urine as opposed to male pigs. Also, “indigenous breed” pigs (OR: 12.40, 95% CI: 5.15–29.82) pigs were significantly more likely to have AFM_1_ in their urine compared to “exotic breed” pigs (Table 3).

**Table 3:** Logistic regression model of AFM_1_ (positive was considered if above the limit of detection, ≥ 0.15 μg/kg) for each category of pigs

Variable	Category	Adjusted Odds ratio	95% CI	*P*-value
Sex				
	Male	Reference	N/A	N/A
	Female	1.45	1.08–1.96	0.014*
Breed				
	Exotic	Reference	N/A	N/A
	Indigenous	12.40	5.15–29.82	<0.001*
	Mixed	1.51	0.36–6.32	0.576

*CI* confidence interval, *NA* not applicable as reference group

*= statistically significant at *p* < 0.05

A total of 252 people were interviewed from five provinces (Hanoi: *n* = 49, Son La: *n* = 50, Nghe An: n = 50, Dak Lak: *n* = 53 and An Giang: n = 50) to assess their awareness of aflatoxins. Overall, we found that 67.86% (95% CI: 61.71–73.58) of people were aware of aflatoxins. In addition, age groups 21–29 and 30–39 had relatively high awareness of aflatoxins whereas those aged under 20 and those between 50 and 59 years of age had the lowest awareness of aflatoxins (Table 4).

**Table 4:** Demographic characteristics of survey respondents from “*Have you heard about aflatoxins?”*

Category	Characteristic (n)	Have you heard about aflatoxins?
Age	< 20 (n = 3)	1 (33.33%)
(year)	21–29 (*n* = 21)	16 (76.19%)
	30–39 (*n* = 65)	46 (70.77%)
	40–49 (*n* = 89)	62 (69.66%)
	50–59 (*n* = 54)	32 (59.26%)
	≥ 60 (*n* = 20)	14 (70.0%)
Gender	Male (*n* = 154)	114 (74.03%)
	Female (*n* = 98)	57 (58.16%)
Education	None (n = 3)	1 (33.33%)
	Primary & Middle school (*n* = 97)	49 (50.52%)
	High school (*n* = 115)	88 (76.52%)
	College/University or more (*n* = 37)	33 (89.19%)
Occupation	Farmers (*n* = 141)	90 (63.83%)
	Retailers (*n* = 36)	27 (75.0%)
	Feed manufacturers (*n* = 10)	9 (90.0%)
	Others (office workers and businessmen) (*n* = 65)	45 (69.23%)

Gender and education were significantly associated with awareness in the univariable analysis and were therefore included in the model. Our final model showed that male (OR: 2.64, 95% CI: 1.15–6.09), “High school” group (OR: 3.40, 95% CI: 1.69–6.86) and “College/university or more” group (OR: 10.20, 95% CI: 4.54–22.89) had significantly increased awareness of aflatoxins compared to the reference group (female and primary/secondary school group) (Table 5). The Hosmer-Lemeshow goodness of fit test showed that there was no evidence of poor fit (*p*-value = 0.27).

**Table 5:** Final multivariable logistic regression model of awareness of aflatoxins in five provinces in Vietnam

Variable	Category	Odds ratio	95% CI	*P*-value
Gender				
	Female	Reference	N/A	N/A
	Male	2.64	1.15–6.09	0.022*
Education				
	None	0.34	0.03–3.91	0.389
	Primary & Secondary school	Reference	N/A	N/A
	High school	3.40	1.69–6.86	0.001*
	College/university or more	10.20	4.54–22.89	<0.001*

*CI* confidence interval, *NA* not applicable as reference group

*= statistically significant at *p* < 0.05

## Discussion

This was the first national study to systematically evaluate the concentrations of AFM_1_ in the urine of pigs in Vietnam. We found that samples from Son La had the highest positive rate; this was consistent with previous research on maize samples which found that Son La had a higher proportion of samples with AFB_1_ above 2 and 5 μg/kg, compared to five other provinces [30]. Son La is one of the most important maize production areas in Vietnam, mainly due to its suitable agro-climatic conditions and high altitude [37]. Maize is an important source of income for ethnic minorities as well as being one of their staple foods. Moreover, it is the main source feed for livestock (such as pigs and cattle) in Vietnam. In humans and animals, aflatoxins in urine have been used as a marker of exposure, and it has been shown that there is a correlation between aflatoxin intake and serum concentrations, as well as serum concentrations and urine AFM_1_ concentrations [32, 38]. Therefore, a rigorous investigation is necessary to assess the full impact of AFB_1_ and AFM_1_ on plant, animal and human health in Son La province.

Overall, significantly higher positive rates in the northern region (Hanoi and Son La) were observed compared to the southern region (Dak Lak and An Giang), which may be attributed to differences in climatic conditions. In Vietnam, the northern and southern regions are classified into subtropical and tropical conditions, respectively. Some studies have suggested that climatic conditions, particularly temperature and humidity, affect *Aspergillus* growth and aflatoxin production [39–42], and it is possible that this also had an influence on the regional differences observed in this study. Processing also affects aflatoxin production, with insect damaged and high moisture corn being major predisposing factors for contamination. Based on our work, livestock in Vietnam are exposed to AFB_1_, and there is a risk that meat, eggs and dairy products contain AFM_1_. This is because the feed used in these livestock systems is very similar and contains a large proportion of maize. Although few studies have been conducted on dairy products, one study in Ho Chi Minh City found that 32.6% milk samples contained AFM_1_ while one of 46 samples exceeded the limit (0.05 μg/L) under Vietnamese regulation [43]. However, there is currently no guidance on AFB_1_ levels in animal feed under the Vietnamese regulations, while 5 μg/kg is commonly used as the tolerated level for feedstuffs for dairy cattle in European Union countries [44]. The United States Food and Drug Administration (FDA) guidelines for total aflatoxin levels are between 20 μg/kg and 300 μg/kg, depending on the commodities and intended species [45]. For corn, less than 20 μg/kg is considered safe for use in all animal feed. Exposure to aflatoxins has adverse effects on human and animal health. Therefore, future research is needed to assess the potential adverse effects of AFM_1_ residues in meat and dairy products in Vietnam as well as to establish regulations for animal feed to reduce the negative health impacts in humans and animals.

The main limitation of this study was that most of our samples were collected during the dry season, yet aflatoxin levels are seasonally heterogeneous. In a study in Sierra Leone, AFM_1_ levels from urine samples in humans were higher during the rainy season than the dry season [46]. Another potential bias is that clinically healthy pigs are over-represented as they are more likely to be slaughtered. Although neither slaughterhouses nor participants in the survey were selected probabilistically, we did not expect much bias to have been introduced here, and these samples to be representative.

We found that female pigs had significantly higher exposure to AFM_1_, although fattening pigs may be expected to be fed the same type of feed independent of gender. However, male animals are known to be more susceptible to aflatoxins than females [47, 48] and it is possible that females could have a higher clearance of aflatoxins through urine, and thus be more protected against the harmful effects. Even among the two sows studied by Lüthy et al. [31], there was a difference in the proportion of fed AFB_1_ that was excreted as AFM_1_, 9.6% and 15.7% respectively, so difference in excretion among pigs may be possible. If pregnant and suckling sows are exposed to aflatoxins, this could negatively impact productivity as some studies have shown that chronic exposure to aflatoxins led to lower growth rate in piglets [49, 50]. Urine concentrations of AFM_1_ were higher in indigenous pigs but the reason for this is unclear. Indigenous pigs are more likely to be raised by ethnic minorities who are less educated and thus have low levels of awareness of aflatoxins, increasing the chances of the animals consuming feed with high levels of AFB_1_. Moreover, susceptibility to aflatoxins varies by breed and this may play a role.

Aflatoxin exposure in pigs has multiple implications. From the food safety point of view, aflatoxins carried over to pork are a food safety hazard. Given that pork accounts for about 70% of livestock production in Vietnam [51], this could be a significant source of human exposure. Pork is of concern as a possible exposure route as it is consumed by more than 95% of the population in Vietnam, with an annual consumption of approximately 24.7 kg per capita [52]. From the farm management point of view, the control of aflatoxin contamination needs to be considered for animal health and economic benefits. In addition further studies are needed to confirm if differences in urine aflatoxin levels are due to differences in exposure or clearance, although human studies have used it as an exposure assessment [38].

Our survey showed that men and more educated groups were more aware of aflatoxins. It is well recognized that Vietnamese women are undervalued and it is not uncommon for women to have limited access to higher education and suffers from lower pay in occupational sectors [53, 54]. The age groups less than 20 and 5–59 showed that awareness of aflatoxins relatively lower while it is worthwhile to conduct further investigation why awareness was low for these groups. It is recommended that women and less well educated groups are targeted for raising the public awareness of aflatoxin risks as well as introduction of control and prevention strategies. The intervention programs may include timing of planting, avoiding drought and rodent/insect control for field management, washing, rapid and proper drying and cleaning for post-harvest [55].

## Conclusion

We can conclude that pigs in Vietnam are exposed to aflatoxins to varying degrees, and there may be a risk that pork products could contain AFM_1_. Further investigation is needed into the possible health impacts as well as to aid in establishing regulations for animal feed to reduce the health impacts in humans and animals.

## Abbreviations

AFB_1_: Aflatoxin B_1_
AFM_1_: Aflatoxin M_1_
CI: Confidence interval
ELISA: Enzyme-linked immunosorbent assay
FDA: Food and Drug Administration
IARC: International Agency for Research on Cancer
LOD: Limit of detection
NIVR: National Institute of Veterinary Research
OD: Optical density
OR: Odds ratio
